# IL-33/ST2 axis is involved in disease progression in the spleen during *Leishmania donovani* infection

**DOI:** 10.1186/s13071-020-04190-3

**Published:** 2020-06-22

**Authors:** Aurore Lamberet, Octavie Rostan, Sarah Dion, Aurélien Jan, Hélène Guegan, Christelle Manuel, Michel Samson, Jean-Pierre Gangneux, Florence Robert-Gangneux

**Affiliations:** 1 Inserm, EHESP, IRSET (Institut de Recherche en Santé Environnement et Travail)-UMR_S 1085, CHU Rennes, University of Rennes, 35000 Rennes, France; 2 Inserm, EHESP, IRSET (Institut de Recherche en Santé Environnement et Travail)-UMR_S 1085, University of Rennes, 35000 Rennes, France

**Keywords:** Visceral leishmaniasis, *Leishmania donovani*, IL-33, Spleen, Immune response, ST2

## Abstract

**Background:**

During infection with *Leishmania donovani*, parasite control is linked to the systemic Th1 immune response, but in infected organs (liver, spleen and bone marrow), the response differs according to the micro-environment. The pleiomorphic cytokine interleukin-33 (IL-33) exerts various roles during infection, either protective or detrimental. In this study, we explored the role of IL-33 in the outcome of *Leishmania* infection in the spleen.

**Methods:**

We used several mouse models, on BALB/c and C57BL/6 (B6) backgrounds, infected with *L. donovani* and sacrificed at 15, 30 or 60 days after infection and characterized mRNA expression of immune markers, immune cell populations, histological response, and parasite loads.

**Results:**

During infection IL-33 and ST2 mRNA increased in parallel in the spleen of wild type (wt) animals and paralleled the immunodetection of ST2+ and IL-33+ cells; their expression was twice as high in BALB/c, compared to B6 mice. Mice treated with twice-weekly injections of rIL-33 had higher splenic parasite burdens on D15 (BALB/c) or on D60 (B6). In BALB/c, IL-33 treatment led to immune exhaustion with abolition of Th1 cytokine expression (IFN-γ and IL-12) in the spleen and higher serum levels of Th2 cytokines (IL-4, IL-5 and IL-13). In B6, IL-33 treatment induced the Treg cell pathway with a dramatic increase of FoxP3 mRNA induction and expression on tissue sections. IL-33-KO mice had lower parasite loads and a higher Th1 response than their wt counterparts.

**Conclusions:**

IL-33 appears as a factor of aggravation of the disease in the spleen tissue of mice infected with *L. donovani.*
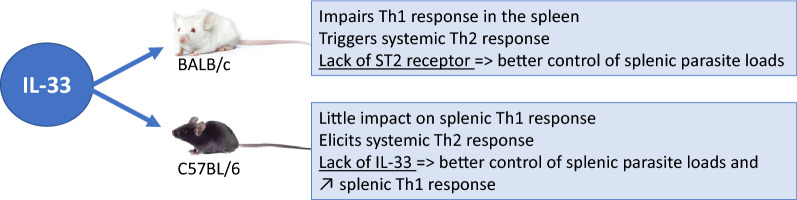

## Background

Visceral leishmaniasis (VL) is a systemic life-threatening infection, due to the intracellular protozoans *Leishmania donovani* and *L. infantum.* VL is present in 92 countries worldwide with an estimated incidence of 400,000 cases per year, resulting in approximately 30,000 deaths annually [1]. Leishmaniasis is ranked by the World Health Organization as second most important protozoan parasitic disease after malaria for its morbidity, mortality and global distribution. After inoculation of the promastigote forms of *Leishmania* by the sand fly vector, parasites are passively phagocytosed by phagocytic cells, mainly macrophages, which are the key target cells for amastigote replication, but also polymorphonuclear cells. Macrophages deliver the parasites to the main target organs, i.e. the spleen, the bone marrow and the liver. The liver is the site of an active tissue response which leads to the decrease of parasite burden *via* the formation of granulomas. Infection control is known to be linked to an efficient Th1 immune response, while a Th2 response is associated to progression of disease. However, a mixed Th1/Th2 response is observed specifically in the liver during VL, which warrants a granulomatous tissue response ensuring the control of parasitic loads in this organ [2, 3]. By contrast, uncontrolled parasite proliferation is observed in the spleen, as shown in mouse models, causing splenomegaly and persistence of amastigotes [4]. The role of the pro-inflammatory Th2 interleukin-33 (IL-33) has been investigated in various infectious diseases, where it was shown to have either a protecting effect against severe manifestations or a deleterious role favoring disease progression [5, 6]. Our team has previously reported elevated concentrations of IL-33 in the serum of patients infected with *L. infantum*, and showed in a mouse model of *L. donovani* infection, that IL-33 was associated with poorer control of infection in the liver [5]. The role of IL-33 in parasite persistence in the spleen is not documented to date. As the respective microenvironment in the liver and the spleen are very different, results obtained in the former cannot predict the role of this cytokine in the latter. IL-33 is associated with cell signaling *via* the ST2 receptor, which is present on the surface of many cells: Th2 cells, invariant NKT, NK, cytotoxic T lymphocytes, monocytes, macrophages, dendritic cells, neutrophils and endothelial cells, explaining the various effects of this cytokine [6, 7]. Therefore, the aim of this study was to characterize the impact of IL-33 on the splenic tissue response during VL, using various mouse models to better understand the effects of this inflammatory cytokine known to have contrasting effects according to patient background and disease [6, 8, 9].

## Methods

### Mice

Female BALB/c and C57BL/6 wild-type (wt) mice were purchased from Janvier Laboratories (Le Genest-Saint-Isle, France) and acclimatized for at least 10 days before challenge. BALB/c ST2-KO mice [10] and C57BL/6 IL-33-KO mice were backcrossed for at least 10 generations. ST2-KO mice were originally obtained from Dr Andrew McKenzie (MRC Laboratory of Molecular Biology, Cambridge, UK) and IL-33-KO mice were kindly provided by Jean-Philippe Girard (Université de Toulouse, Toulouse, France). Mice were bred and housed in our animal facilities. Mice were 7 to 10 weeks-old when challenged with *L. donovani*. Naïve congenic mice, matched according to age, were used as non-infected controls. The results were obtained in two to three independent experiments, with a total of 5 to 13 mice per time point.

### Parasites and infection of mice

The *L. donovani* strain (MHOM/SD/97/LEM3427, typed as Zym MON-18 by the Centre National de Référence des Leishmanioses, Montpellier, France) was maintained *in vivo* by serial murine passages and grown *in vitro* on home-made Novy-McNeal-Nicolle blood agar at 27 °C. Prior to infection, amplification of promastigotes was carried out by culture in Schneider’s *Drosophila* medium (Invitrogen, Carlsbad, CA, USA) supplemented with 10% FCS, 100 U/ml penicillin and 100 µg/ml streptomycin, for 6 days at 27 °C, until they reached stationary phase. Animals were infected on day 0 (D0) by intraperitoneal injection of 10^8^ promastigotes, and groups of mice were sacrificed on D15, D30, or D60. Prior to sacrifice, blood was collected by retro-orbital puncture, and the serum was stored at − 80 °C. The spleen was recovered and weighed, cut into pieces and then used for immune cell typing by flow cytometry or formalin-fixed and paraffin-embedded or snap frozen in isopentane/liquid nitrogen for mRNA extraction.

### Treatment with recombinant IL-33

Recombinant IL-33 (rIL-33) was purchased from Peprotech (Neuilly-sur-Seine, France). Mice (4–5 animals per time point) were infected with *L. donovani* at D0 as previously described [5] and treated by intraperitoneal injection of 0.5 µg of rIL-33 per mouse twice a week until their sacrifice at D15, D30 or D60. Non-treated BALB/c and C57BL/6 mice were used as controls and infected with the same parasite inoculum.

### Quantification of spleen parasitic burden

The parasitic loads were quantified by quantitative PCR using a Touch™ q-PCR system (Bio-Rad, Marnes-la-Coquette, France) by amplification of the *18S* gene specific of *L. donovani* and comparison to a standard curve (0.1–10^6^ *L. donovani*/ml). The parasitic load was then reported to the weight of the organ and expressed in mg of spleen.

### Histological quantification of germinal centers areas

Paraffin-embedded tissue was cut at 4 µm, mounted on slides and dried at 58 °C for 60 min. Hematoxylin Eosin Safran (HES) staining was performed to reveal the architecture of the spleen. The slides were scanned using a scanner (Nanozoomer 2.ORS; Hamamatsu, Massy, France) and areas of germinal centers (GC) were quantified using the NDP view 2.4.26® software (Hamamatsu).

### Immunohistochemical characterization of immune cells in the spleen

Immunohistochemical studies were performed as previously described [11]. Paraffin-embedded tissue was cut at 4 µm, mounted on positively charged slides and dried at 58 °C for 60 min. Immunohistochemical staining was performed on the Discovery Ultra Automated IHC stainer using the Ventana DABMap® detection kit (Ventana Medical Systems, Tucson, USA). Antigen retrieval was performed using Ventana proprietary, Tris-based buffer solution CC1 at 95 °C to 100 °C for 36 min. Endogen peroxidase was blocked with inhibitor-D 3% H_2_O_2_ (Ventana) for 8 min at 37 °C. After rinsing, slides were incubated at 37 °C for 60 min with primary goat anti-mouse IL-33 diluted to 1/50 (R&D systems, Noyal Châtillon sur Seiche, France) or rat anti-mouse ST2 antibody diluted 1/100 (DJ8; MB Bioproducts, Zurich, Switzerland) or rat anti-FoxP3 (R&D systems) and respective secondary antibodies, biotinylated anti-goat or anti-rat for 30 min. Signal enhancement was performed using the Ventana DABMap^®^ Kit. Slides were then counterstained for 16 min with hematoxylin and rinsed. After removal from the instrument, slides were manually dehydrated and mounted with a coverslip. Appropriate controls were made to validate the antibodies; no staining was observed without primary antibodies, and ST2-KO or IL-33-KO mice showed no ST2 or IL-33 staining, respectively.

### Detection of cytokines in plasma

Murine cytokines were quantified by bead-based immunoassays adapted on flow cytometry, using the mouse Th1/Th2 10-plex FlowCytomixTM Kit (eBioscience, San Diego, CA, USA) according to the manufacturer’s instructions, using a filter plate and a vacuum filtration system for washing steps. Data were collected on a FC500® cytometer (BD Biosciences, San Jose, USA), and analyzed with FlowCytomix® Pro 3.0 Software (eBioscience). Standard curves were determined for each cytokine from a range of 27–20000 pg/ml. Serum concentrations were expressed in mean fluorescence intensity (MFI).

### RNA isolation and analysis of splenic gene expression

Total cellular RNA was extracted and purified from spleen samples using Trizol reagent (Invitrogen, Thermo Fisher Scientific, Illkirch, France), then treated with DNase (10 U DNase I/µg total RNA) and reverse transcribed with a High Capacity cDNA Reverse Transcription kit (Applied Biosystems, Thermo Fisher Scientific) according to the manufacturer’s instructions. Quantitative PCR amplifications were carried out in duplicate using Power SYBR®green PCR Master Mix (Applied Biosystems), 3 µM primers and cDNA corresponding to 30 ng of total RNA input in a final volume of 10 µl, in 384-well optical plates, using the CFX384 Touch™ q-PCR system (Bio-Rad). The PCR primers were designed using Primer express 3 software and synthesized by Qiagen or Sigma-Aldrich (Lyon, France). Expression levels of target genes were normalized by comparison to expression of *18S* rRNA. Results were expressed as 2^−ΔΔCq^ referred as fold induction in relation to the mean Cq obtained with non-infected wt mice.

### Flow cytometry analysis

After homogenization of spleen tissue, immune cells were purified using 35% Percoll (GE Healthcare, La Chapelle-sur-Erdre, France) and red blood cells were lyzed. 10^6^ leucocytes were incubated with anti-CD16/32 (BD Pharmingen, Rungis, France) to block non-specific binding and washed. Cells were then incubated 30 min with appropriate dilutions of anti-CD3-Pacific Blue, anti-CD8-APC-Cy7, anti-CD4-PE, anti-NK1.1-Percp-Cy5.5 and anti-CD19-APC antibodies, all purchased from BD Pharmingen. The staining of ST2 was assessed with a rat monoclonal anti-mouse ST2-FITC antibody (clone DJ8; MB Bioproducts). Cells were washed, fixed in PBS containing 2% FCS, 0.01 M sodium azide and 2% formaldehyde and analyzed on a FACS Aria II ® flow cytometer using BD FACS Diva software (BD Biosciences). Dead cells and doublet cells were excluded on the basis of forward and side scatter. The different immune cell types were identified and gated as follows: BL were CD19^+^; NK cells were NK1.1^+^/CD3^−^; T CD8^+^ lymphocytes were NK1.1^−^/CD3^+^/CD8^+^/CD4^−^; and T CD4^+^ lymphocytes were NK1.1^−^/CD3^+^/CD4^+^/CD8^−^. The gating strategy was previously described [5, 12].

### Statistical analysis

Data were expressed as mean ± standard error of the mean (SEM) for each group of mice (3–8 mice per group) from 2–3 independent experiments). Differences between groups were analyzed using a two-way ANOVA comparison followed by Bonferroni tests between groups. Selective comparisons were occasionally made using the non-parametric Mann-Whitney test. Correlations between variables were evaluated using the Spearman rank correlation test. Statistical analysis was performed using GraphPad Prism 6 software. Differences were considered significant when the *P*-value was < 0.05, and indicated as *(*P* < 0.05), **(*P* < 0.01) and ***(*P* < 0.001).

## Results

### IL-33 and its receptor ST2 are more expressed in the spleen of susceptible BALB/c mice, compared to C57BL/6 mice

**Fig. 1:**
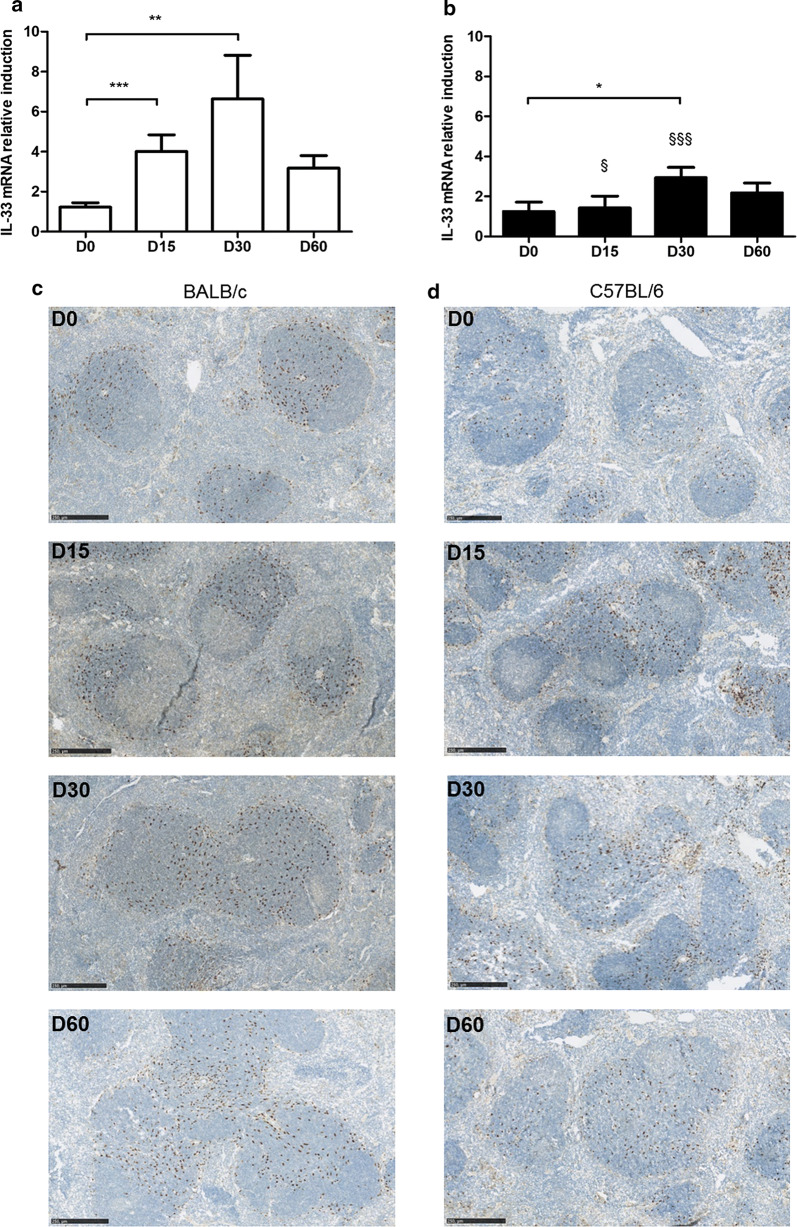
IL-33 expression in the spleen of BALB/c and B6 mice after infection with *L. donovani.* Quantification of IL-33 mRNA induction in spleen biopsies of BALB/c (**a**) and B6 (**b**) mice at various time points following infection with *L. donovani.* Representative data (mean ± SE) of at least two experiments (*P < 0.05, **P < 0.01) (^§^*P* < 0.05, ^§§§^*P* < 0.001 by comparison to BALB/c mice, Mann-Whitney test). Immunohistochemical staining of spleen tissue sections of BALB/c (**c**) and B6 (**d**) mice, using a goat anti-mouse IL-33 antibody. Representative fields observed at 200× magnification by optic microscopy. *Scale-bars*: **c**, **d**, 250 µm

Although the expression of IL-33 mRNA was roughly similar in both mouse strains at baseline, it increased earlier in BALB/c, and twice as much in BALB/c as in C57BL/6 (B6) mice (ANOVA: *F*_(1, 51)_ = 16.97, *P* < 0.001 and *P* < 0.01 at day 15 and day 30, respectively) (Fig. 1a, b). Immuno-histochemical staining of spleen tissue using an IL-33 antibody confirmed that IL-33 expressing cells were present in higher amounts in BALB/c mice than in B6 mice, particularly on Day 15 and Day 30 (Fig. 1c). IL-33+ cells were mainly located in germinal centers in BALB/c mice, irrespective of the time point. In B6 mice, a weak intensity of staining was observed at basal level, and increased slightly in germinal centers on Day 15, with migration of cells onto the marginal zone and diffusion towards the red pulp on Day 30 (Fig. 1d). Overall, the location of IL-33+ cells was similar in both mouse strains.

**Fig. 2:**
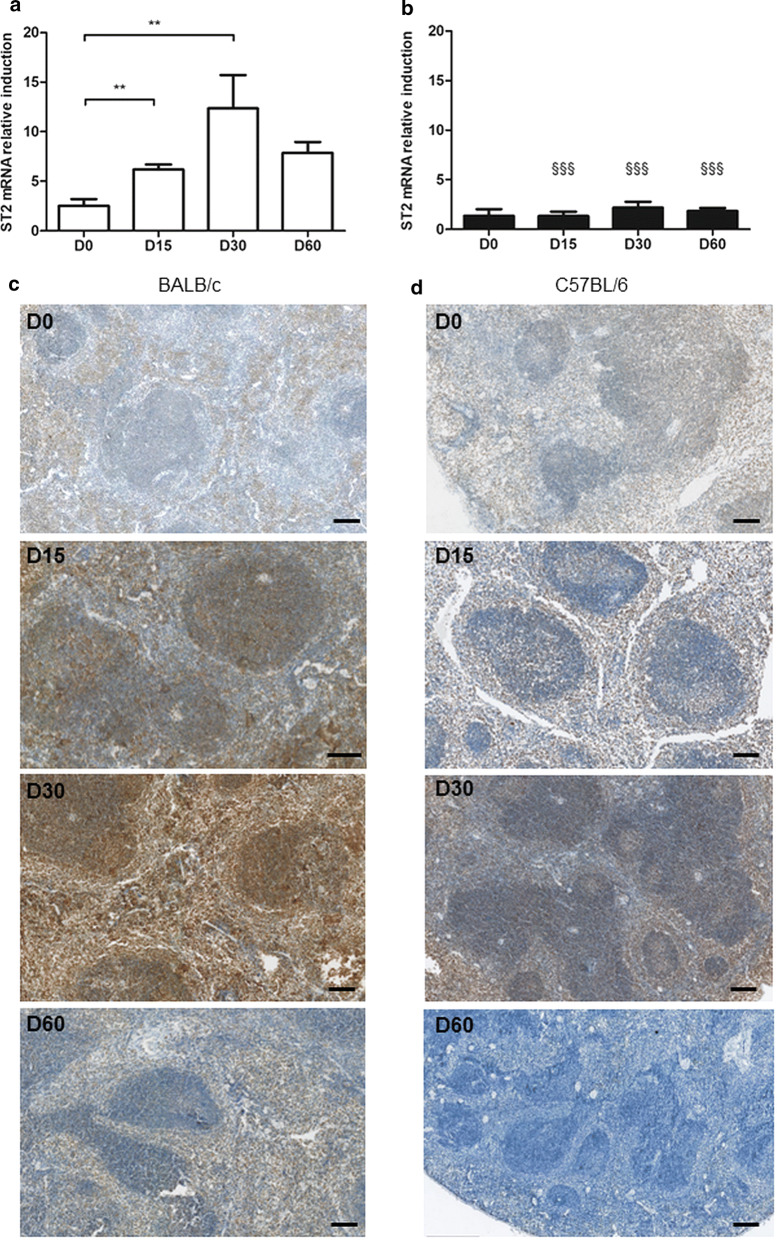
ST2 expression in the spleen of BALB/c and B6 mice after infection with *L. donovani.* Quantification of ST2 mRNA induction in spleen biopsies of BALB/c (**a**) and B6 (**b**) mice at various time points following infection with *L. donovani.* Representative data (mean ± SE) of at least two experiments (**P < 0.01) (^§§§^*P* < 0.001 by comparison to BALB/c mice, Mann-Whitney test). Immunohistochemical staining of spleen tissue sections of BALB/c (**c**) and B6 (**d**) mice, using a rat anti-mouse ST2 antibody. Representative fields observed at a 200× magnification by light microscopy. *Scale-bars*: **c**, **d**, 100 µm

ST2 mRNA expression paralleled that of IL-33 in BALB/c mice, peaking on Day 30 (Mann-Whitney test: *U* = 0.0, *P* = 0.0091, compared to D0) (Fig. 2a), while no significant induction was noted in B6 mice (Fig. 2b). ST2 was mainly distributed on cells of the red pulp in non-infected mice (Fig. 2c, d). The number of ST2^+^ cells increased dramatically after infection in both the red pulp and the germinal centers in BALB/c mice on Day 30, reflecting either an influx of ST2^+^ cells, or a higher expression of the receptor at the cell surface, and returned to a basal level on Day 60 (Fig. 2c). In B6 mice, immuno-histochemical staining showed a similar trend as for BALB/c mice, though to a far lesser extent, and the increase of ST2+ cells was essentially observed in the red pulp (Fig. 2d).

### IL-33/ST2 axis supports lack of parasite control in the spleen

Deficient or supplemented mouse models were used to dissect the role of the IL-33/ST2 axis in the spleen, following *L. donovani* infection. We used BALB/c ST2-KO mice, B6 IL-33-KO mice, and their respective wt littermates. Additionally, B6 mice and BALB/c wt mice were treated with recombinant IL-33 (rIL-33) twice a week until sacrifice on Day 60, as previously described [5].

**Fig. 3:**
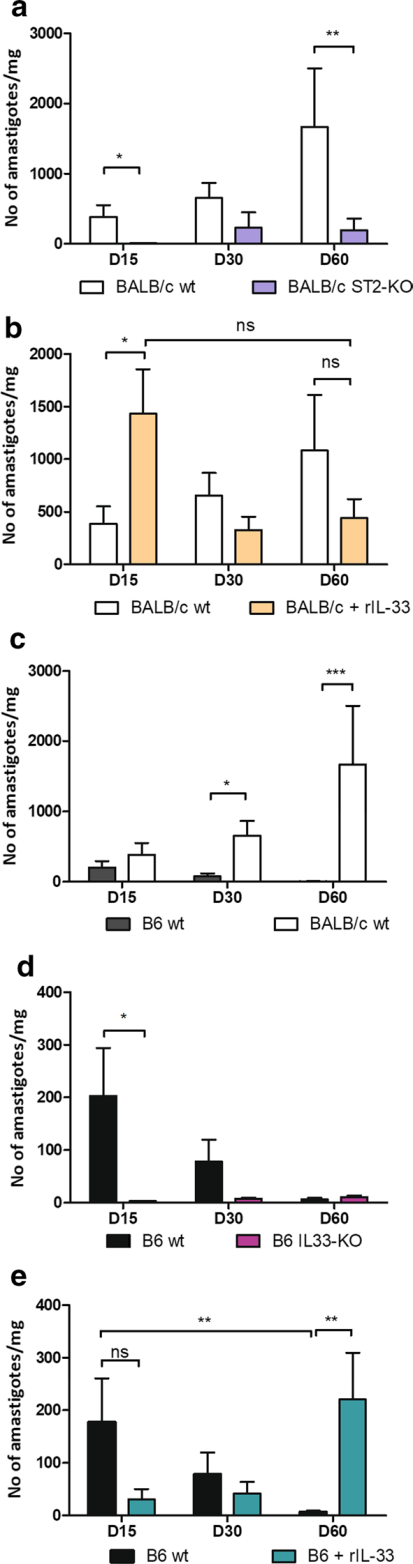
Quantification of *L. donovani* parasite loads in the spleen. Mice were sacrificed at the indicated time points after infection and parasites were quantified by quantitative PCR targeting *Leishmania 18S* RNA and expressed by mg of tissue. Data show means ± SE of parasite loads from representative experiments performed on groups of 3 to 6 mice, comparing BALB/c wt and ST2-KO (**a**), B6 wt and IL-33-KO (**b**), BALB/c wt and B6 wt (**c**), BALB/c treated or not with rIL-33 (**d**) and B6 treated or not with rIL-33 (**e**). *P < 0.05, **P < 0.01, ***P < 0.001; Mann-Whitney test)

Parasite loads increased over time in BALB/c wt, whereas they remained at a low level in ST2-KO mice (Fig. 3a). They were significantly higher in BALB/c wt mice at early time point (Mann-Whitney test: *U* = 20, *P* < 0.05 on Day 15) and on Day 60 (Mann-Whitney test: *U* = 4, *P* < 0.01). Similarly, parasite loads were higher in B6 wt, compared to IL-33-KO mice on Day 15 (Mann-Whitney test: *U* = 1, *P* < 0.05), but they decreased until day 60 to a residual level similar to that observed in IL-33-KO mice, in agreement with better parasite control in B6 mice (Fig. 3b). Of note, striking differences were observed between B6 wt and BALB/c wt on Day 30 (78 ± 42, *versus* 654 ± 215 parasites/mg, respectively, Mann-Whitney test: *U* = 8, *P* < 0.05) and on day 60 (7 ± 3, *versus* 1667 ± 837 parasites/mg, respectively, Mann-Whitney test: *U* = 0.0, *P* < 0.001) (Fig. 3c).

The supplementation with rIL-33 led to discrepant results according to the genetic background. Whereas rIL-33 treatment was associated with an early increase of parasite loads (Mann-Whitney test: *U* = 5, *P* < 0.05 on D15) in BALB/c, they decreased until Day 60, where they appeared lower than in untreated mice, although with no statistical significance (Fig. 3d). In B6, IL-33 injections had little effect on parasite burdens at early time points, but led to a significant increase on Day 60 (Mann-Whitney test: *U* = 1, *P* < 0.01, compared to untreated mice) (Fig. 3e).

### IL-33 is a suppressor of Th1 cytokine response in the spleen

**Fig. 4:**
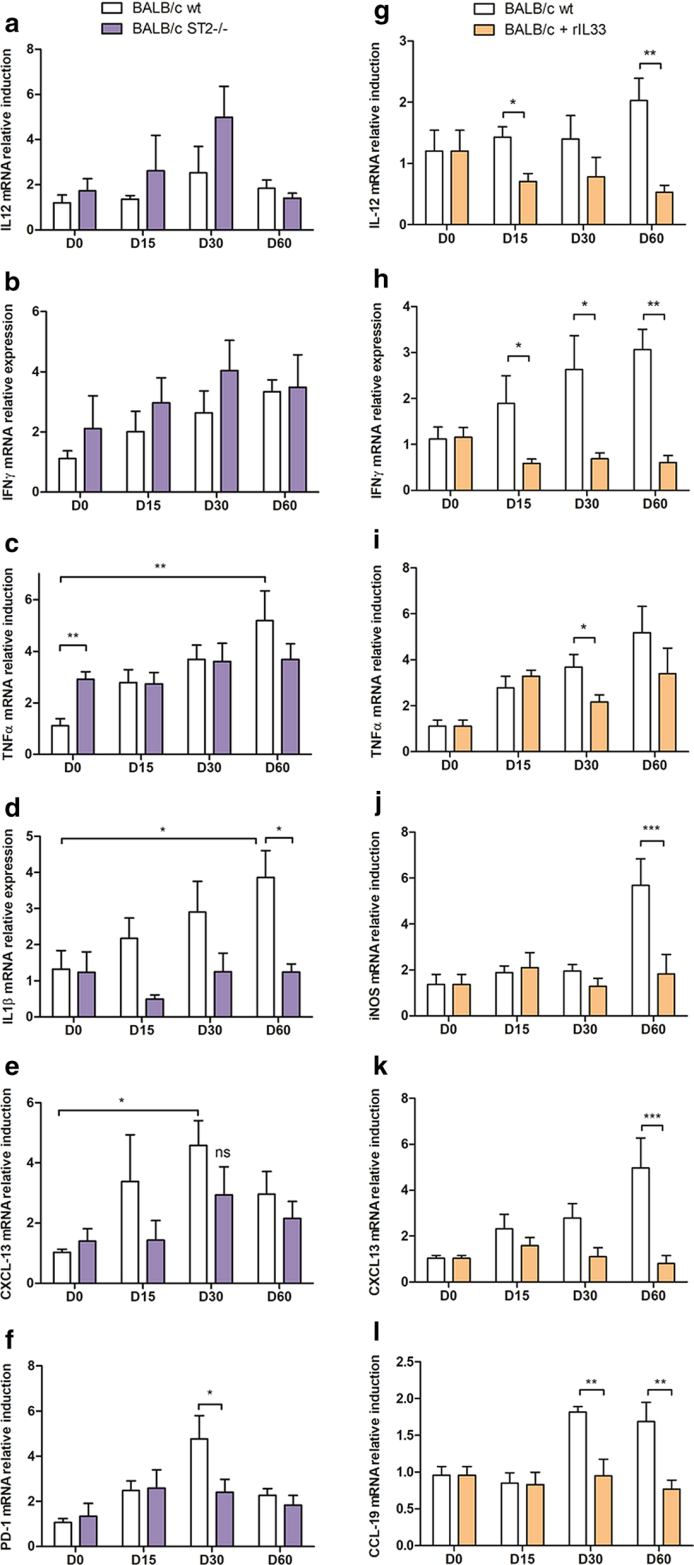
Quantification of mRNA induction of cytokines, chemokines and immune effectors in the spleen of *L. donovani*-infected BALB/c mice. mRNA induction was quantified by quantitative PCR in liver extracts at various time points after infection and normalized by comparison to 18S mRNA. Expression of IL-12 (**a**), IFN-γ (**b**), TNF-α (**c**), IL-1β (**d**), CXCL-13 (**e**), and PD-1(**f**) in BALB/c and ST2-KO mice. Expression of IL-12 (**g**), IFN-γ (**h**), TNF-α (**i**), iNOS (**j**), CXCL-13 (**k**) and CCL-19 (**l**) in BALB/c mice treated with rIL-33 or untreated (wt) (*P < 0.05, **P < 0.01, ***P < 0.001; 2-way ANOVA or Mann-Whitney test)

In BALB/c mice background, the deficiency in the ST2 receptor was associated with a higher expression of TNF-α than in wt animals at a basal level, but no significant differences in IL-12, IFN-γ or TNF-α were observed after infection, between wt and ST2-KO mice (Fig. 4a–c). Interestingly, IL-1β the B lymphocyte-chemoattractant chemokine CXCL-13, and the tolerogenic marker PD-1 were not induced in ST2-KO mice, while they increased significantly until Day 60 (IL-1β or until Day 30 (CXCL-13 and PD-1) in wt (Fig. 4d–f). Cytokine detection in the serum appeared roughly similar in both mouse backgrounds, though there was a trend towards higher levels of IL-1β (Mann-Whitney test: *U* = 3, *P* < 0.05) and CXCL-10 (*P* = 0.06) in ST2-KO mice on Day 30 (Additional file 1: Figure S1a-d).

Repeated treatment with rIL-33 early and sustainably repressed IL-12 and IFN-γ (ANOVA: *F*_(1,37)_ = 11.43 and *F*_(1,41)_ = 13.91, respectively, *P* < 0.01) (Fig. 4g, h). Several other markers (iNOS, CXCL-13, CCL-19, CXCL-10 and IL-10) were also repressed on Day 60, compared to untreated mice (ANOVA: *F*_(3,27)_ = 6.17, *P* < 0.001; ANOVA: *F*_(3,27)_ = 4.11, *P* < 0.001; ANOVA: *F*_(3,21)_ = 4.95, *P* < 0.01, respectively) (Fig. 4i–l, and data not shown). Besides, elevated levels of Th2 cytokines (IL-4, IL-5 and IL-13) were measured in the sera of rIL-33-treated mice on Day 60 (Mann-Whitney test: *U* = 0, *P* < 0.05; Additional file 1: Figure S1e–g).

**Fig. 5:**
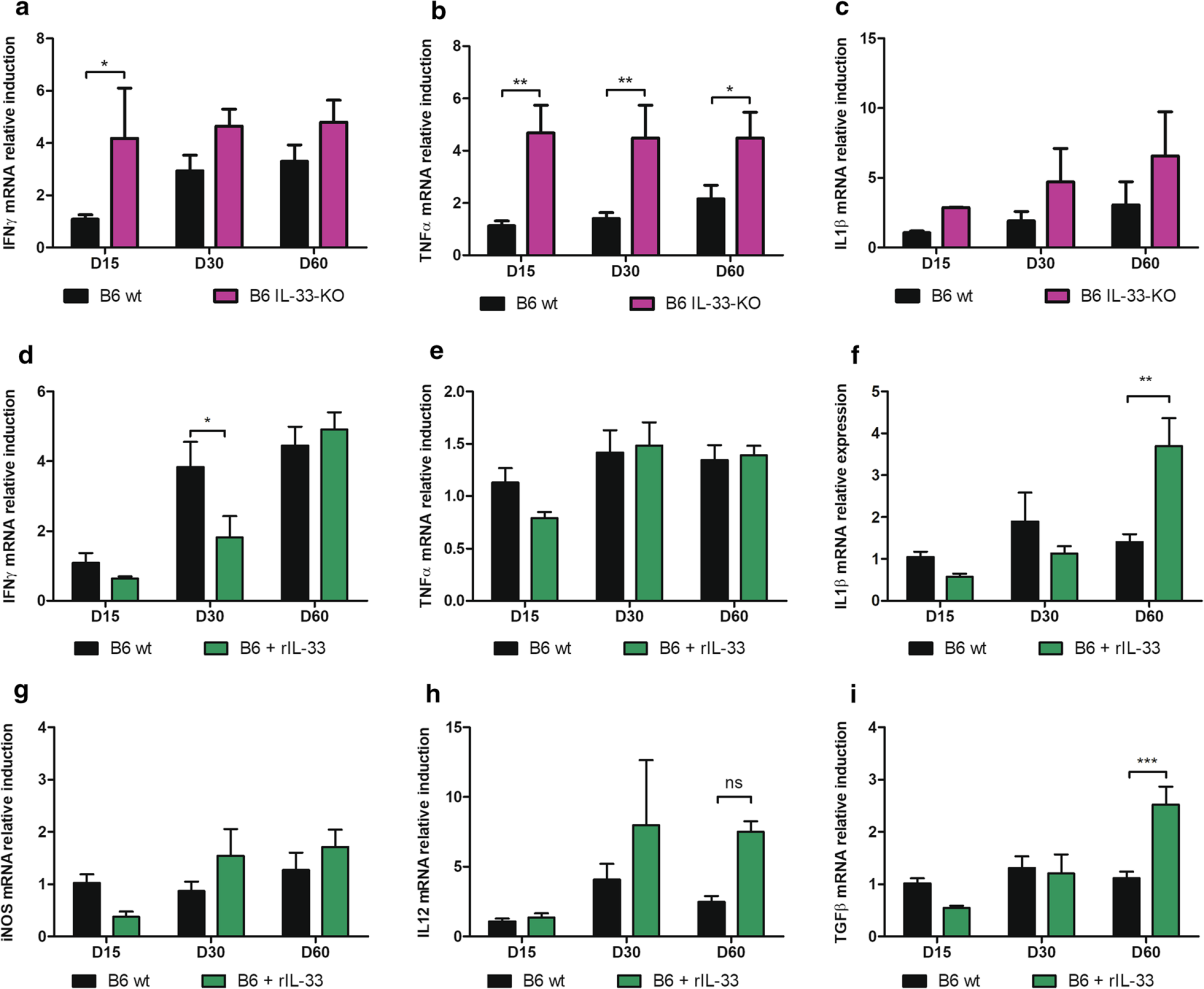
Quantification of mRNA induction of cytokines, chemokines and immune effectors in the spleen of *L. donovani*-infected B6 mice. mRNA induction was quantified by quantitative PCR in liver extracts at various time points after infection and normalized by comparison to 18S mRNA. Expression of IFN-γ (**a**), TNF-α (**b**), IL-1β (**c**), in wt and IL-33 KO B6 mice. Expression of IFN-γ (**d**), TNF- α (**e**), IL-1β (**f**), iNOS (**g**), IL-12 (**h**) and TGF-β (**i**) in B6 mice treated with rIL-33 or untreated (wt) (2-way ANOVA comparison: *P < 0.05, **P < 0.01, ***P < 0.001)

In B6 mice, IFN-γ and TNF-α were significantly more induced in the spleen of IL-33-KO mice than in wt (ANOVA: *F*_(1,27)_ = 11.99, *P* < 0.01 and ANOVA: *F*_(1,30)_ = 33.88, *P* < 0.001, respectively, Fig. 5a, b), whereas in serum, IFN-γ levels were increased only in IL-33-KO mice at D15 (Mann-Whitney test: *U* = 0, *P* < 0.05, Additional file 2: Figure S2a, b). The treatment with rIL-33 did not significantly modify TNF-α nor IFN-γ induction, but increased IL-1β at a late time point (Mann-Whitney test: *U* = 0, *P* < 0.01 on Day 60), and moderately IL-12, while iNOS induction remained unmodified by treatment (Fig. 5d–h). A simultaneous strong induction of TGF-β (Mann-Whitney test: *U* = 0, *P* < 0.01) was also observed on Day 60 in rIL-33-treated mice (Fig. 5i). In rIL-33-treated mice, serum cytokines were detected at very low levels, with a tendency towards higher MFI of Th2 cytokines (IL-5 and IL-13) (Mann-Whitney test: *U* = 0.5 and *U* = 0, respectively, *P* < 0.05, Additional file 2: Figure S2c–e), and lower MFI of inflammatory cytokines (IL-1β, TNF-α and IL-17), in treated mice at Day 15 (Mann-Whitney test: *U* = 0, *P* < 0.05) (Additional file 2: Figure S2f and data not shown).

### IL-33 modifies cell recruitment with differences according to mouse genetic background

**Fig. 6:**
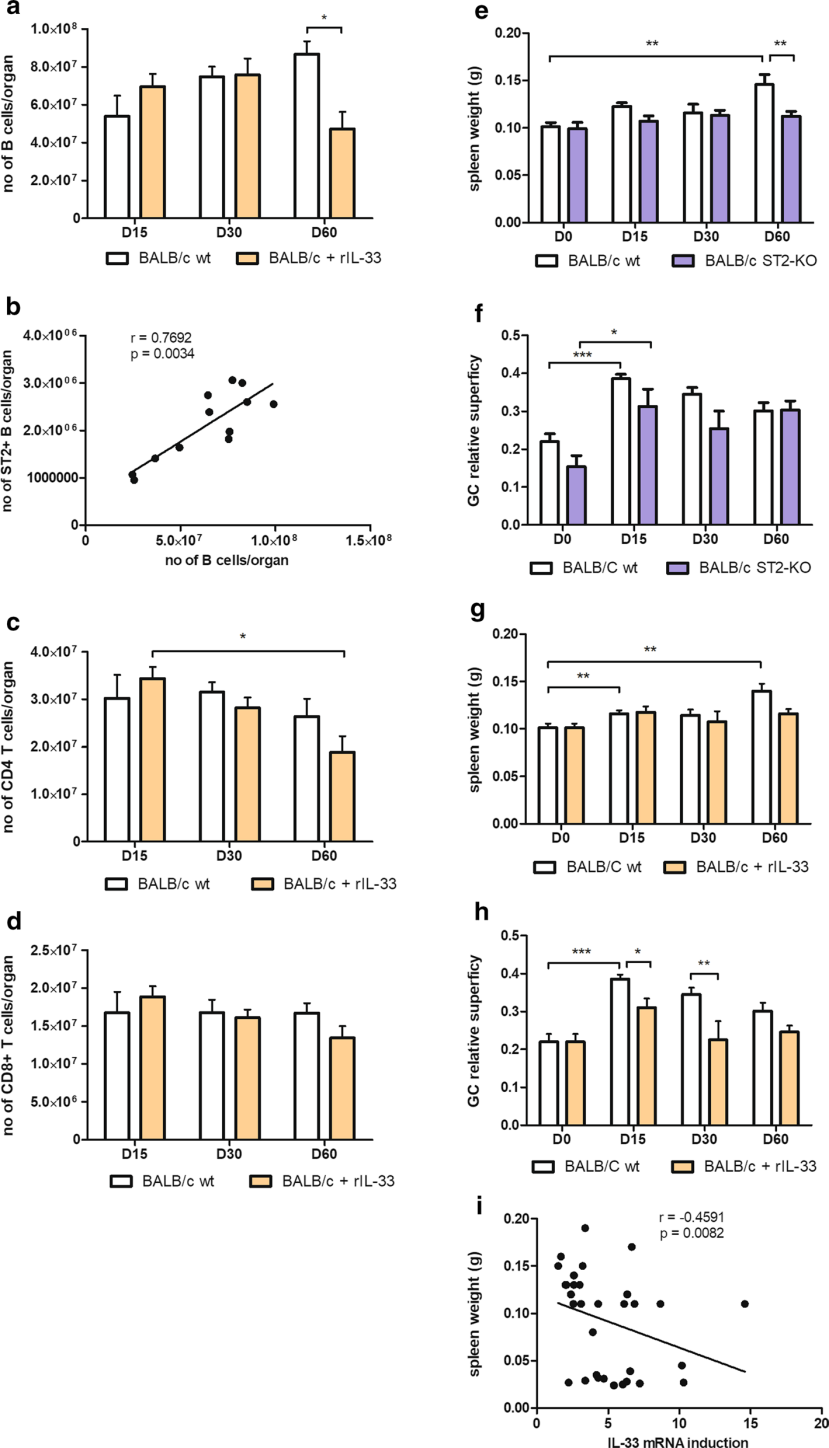
Impact of IL-33 on cell recruitment and histological response in the spleen of BALB/c mice during infection with *L. donovani*. The total numbers of each cell type were quantified by flow cytometry, in mice treated or not with rIL-33 (**a**–**d**). Quantification of B lymphocytes (CD19+) (**a**) and Spearman correlation of the number of B lymphocytes and ST2+ B lymphocytes (**b**), quantification of CD3+/CD4+ T cells (**c**), and CD3+/CD8+ T cells (**d**) per organ. Data are expressed as mean ± SE for each group of mice (4–5 mice per treatment group for each time point) (*P < 0.05; 2-way ANOVA comparison). Spleen weight at indicated time post-infection in BALB/c wt and ST2-KO mice (**e**) and BALB/c mice treated with rIL-33 or untreated (wt) (**g**). Quantification of germinal centers (GC) areas by microscopy using NDP view 2.4.26® software in BALB/c wt and ST2-KO mice (**f**), BALB/c mice treated with rIL-33 or untreated (wt) (**h**). Data are expressed as mean ± SE for each group of mice (4–5 mice per group for each time point) (*P < 0.05, **P < 0.01, ***P < 0.001, with 2-way ANOVA comparison between groups of mice and Mann-Whitney test between time points). Splenomegaly was inversely correlated to IL-33 mRNA expression in BALB/c wt (Spearman correlation)

In BALB/c wt animals, the evolution of disease was associated with an increase of B lymphocyte cells (Fig. 6a), which paralleled splenomegaly (Fig. 6e). Interestingly, the number of ST2^+^ B cells was correlated to the total number of B cells recruited in the spleen (Spearman correlation: *r* = 0.77, *P* < 0.01) (Fig. 6b). The total number of B lymphocytes was also correlated to PD-1 mRNA expression (*P* < 0.001, Spearman correlation, data not shown). No significant change in T cells were observed in wt BALB/c (Fig. 6c, d). By contrast, in ST2-KO mice, the organ weight remained stable over time, and the GC superficies as well, save an early increase following infection, as observed in wt (Fig. 6e, f).

Contrasting with untreated BALB/c mice, the number of B cells decreased on Day 60 after treatment with rIL-33 (ANOVA: *F*_(2, 17)_ = 5.4, *P* < 0.05) (Fig. 6a), and T CD4+ lymphocytes decreased over time (ANOVA: *F*_(2, 17)_ = 6.49, *P* < 0.05) (Fig. 6c), consistent with the reduced area of germinal centers (GC) and the relatively stable spleen weight (Fig. 6g, h). This observation is reinforced by the fact that IL-33 induction was inversely correlated to splenomegaly in untreated animals (Spearman correlation: *r* = − 0.46, *P* < 0.01, Fig. 6i). In rIL-33-treated mice, the GC surface was correlated to the total number of CD4^+^ T lymphocytes (*P* < 0.05, Spearman correlation, data not shown), but not of CD8^+^ T cells. This decrease of cell attraction paralleled the mRNA reduced induction of CXCL-13 and CCL-19 chemokines (Fig. 4).

**Fig. 7:**
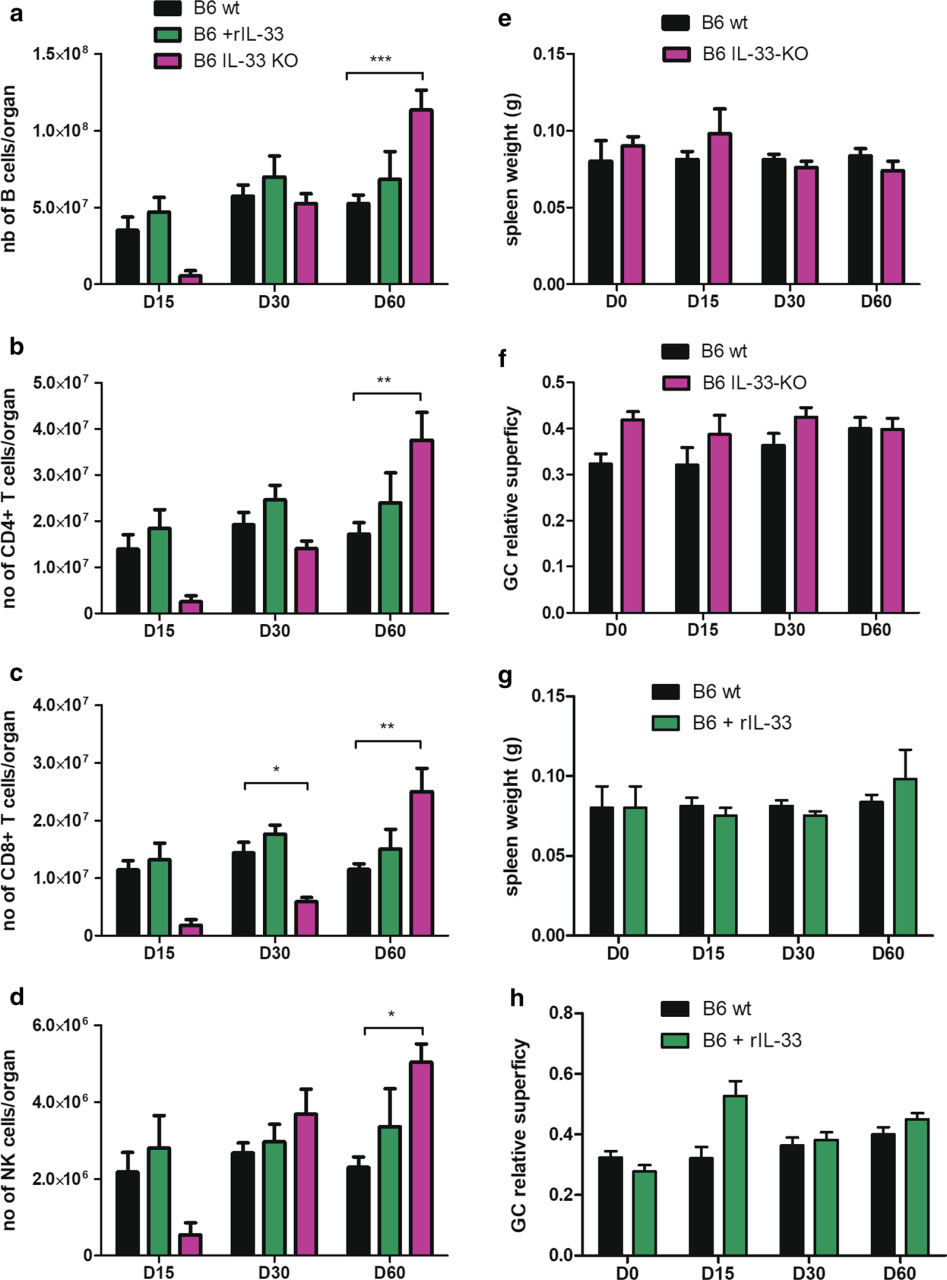
Impact of IL-33 on cell recruitment and histological response in the spleen of B6 mice during infection with *L. donovani.* The total number of B lymphocytes (**a**), CD4^+^ T cells (**b**), CD8+ T cells (**c**) and NK cells (**d**) were quantified by flow cytometry, in mice treated or not with rIL-33 and in IL-33 KO mice. Spleen weight at indicated time post-infection in B6 wt and IL-33 KO mice (**e**) and B6 mice treated with rIL-33 or untreated (wt) (**g**). Quantification of germinal centers (GC) superficies by microscopy using NDP view 2.4.26® software in B6 and IL-33 KO mice (**f**), B6 mice treated with rIL-33 or untreated (wt) (**h**). Data are expressed as mean ± SE for each group of mice (4–5 mice per group for each time point) (*P < 0.05, **P < 0.01, ***P < 0.001; 2-way ANOVA comparison)

In B6 mice, treatment with rIL-33 had little quantitative impact on B and T lymphocyte populations, nor on NK cells (Fig. 7a-d), nor on spleen weight (Fig. 7e), while only a transient hypertrophy of germinal centers was noticed at D15 after infection (Fig. 7f). By contrast, infection in IL-33 KO mice was associated with a significant attraction of B lymphocytes, CD4^+^ and CD8^+^ T lymphocytes, as well as NK cells on Day 60 (Fig. 7a–d), without any clear impact on spleen weight, nor on GC area (Fig. 7g, h), yet ensuring parasite control (Fig. 2b).

**Fig. 8:**
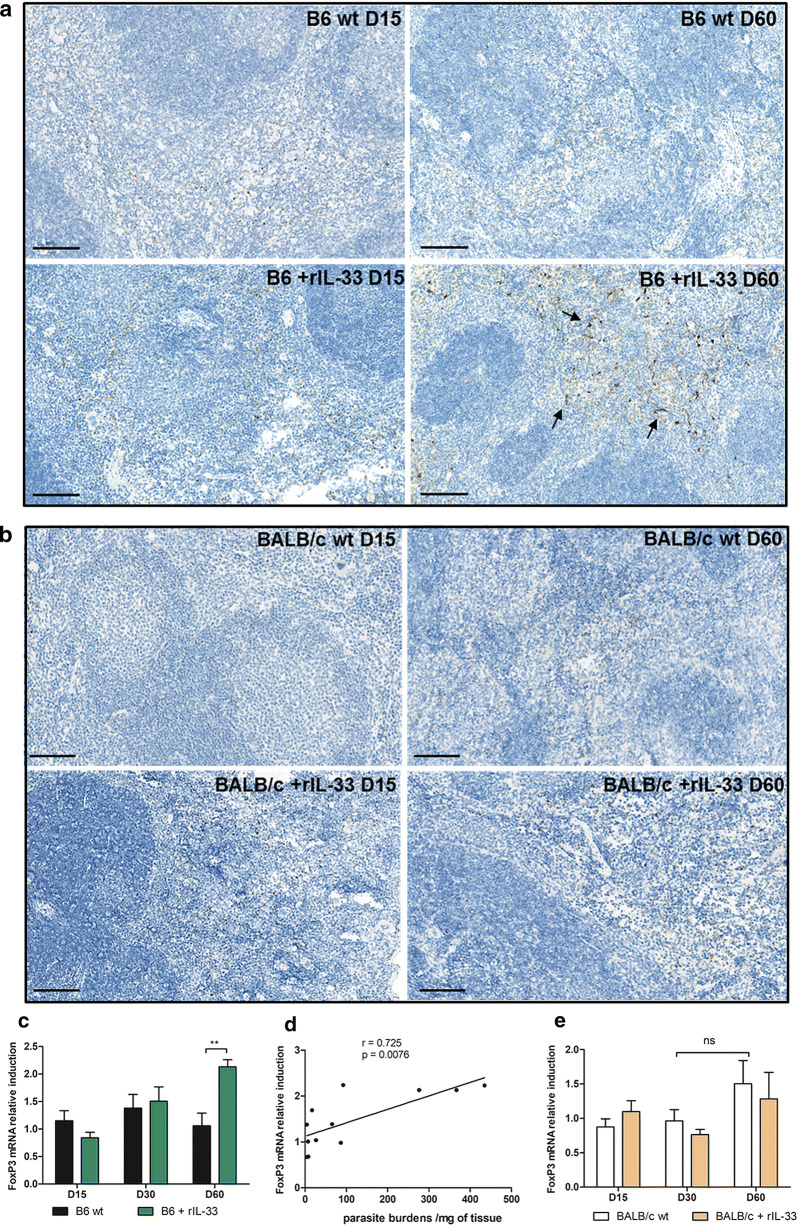
Expression of FoxP3 in the spleen during *L. donovani* infection in mice treated or not with rIL-33. Immunohistochemical staining of spleen tissue sections of B6 (**a**) and BALB/c (**b**) mice using an anti-FoxP3 antibody, at the indicated time points in treated and untreated mice with rIL-33. Representative fields observed at 200× magnification by optic microscopy. Quantification of FoxP3 mRNA induction in spleen biopsies at various time points following infection of B6 (**c**) and BALB/c (**e**) in treated and untreated mice. Representative data (mean ± SE) of at least two experiments (**P < 0.01, 2-way ANOVA comparison). FoxP3 mRNA induction was highly correlated to splenic parasite burdens in B6 mice (Spearman correlation: *P* < 0.01) (**d**). *Scale-bars*: **a**, **b**, 250 µm

To further explore whether the decrease of Th1 cytokines and the apparent lack of cell attraction in IL-33-treated animals could be related to T reg cells, we characterized FoxP3 expression in treated and untreated animals from both genetic backgrounds. On tissue sections, a strong induction of FoxP3^+^ cells was observed in rIL-33-treated B6 mice, by comparison to rIL-33-treated BALB/c mice (Fig. 8a, b). Quantification of mRNA induction of FoxP3 during infection, yielded similar results, with only a significant increase in B6 mice treated with rIL-33 (Mann-Whitney test: *U* = 3, *P* < 0.01). The level of FoxP3 induction was correlated with the parasite loads in the spleen (Spearman correlation: *r* = 0.725, *P* < 0.01) (Fig. 8c–e).

## Discussion

We have previously shown that IL-33 can alter parasite control in the liver [5]. Since liver and spleen behave very differently following *L. donovani* infection, as shown by transcriptomic analysis [13], these first results could not predict findings in the spleen. In the present study, we confirmed that IL-33 also had a deleterious effect on infection control in the spleen, as both IL-33 and ST2 receptor deficiency resulted in better control of parasite burdens in the spleen of mice infected with *L. donovani*, irrespective of the genotype background. However, IL-33 and ST2 were less expressed at the basal level and during infection in B6 wt mice, compared to BALB/c wt mice. Given the fact that ST2^+^ cells are predisposed to respond with a Th2 phenotype [7], this observation could, at least partly, explain the higher sensitivity of BALB/c mice to infection and their Th2-biased phenotype. Not surprisingly, our results showed higher parasite loads in the spleen of BALB/c mice than in that of B6 mice after infection. Also, BALB/c and B6 mice responded differently to repeated injections of rIL-33, with an early increase of parasite loads in BALB/c and a late increase in B6.

In BALB/c wt mice, a significant increase of spleen weight was observed as soon as Day 15 post-infection, which was correlated to an extension of GC areas, but as splenomegaly progressed, GC became more disorganized and reduced in size. Changes in the spleen architecture is a hallmark of visceral leishmaniasis, which induces disorganization of the white pulp and involution of germinal centers, while the red pulp becomes hypertrophied [14, 15], in relation with neovascularization [16]. Here, the relative area of GC were inversely correlated to the expression of IL-33, indicating that it did not contribute to the follicular response, or, alternatively, that it was downregulated when the white pulp grew in size, which is consistent with the decrease of IL-33 expression observed on Day 60. While IL-33+ cells were mainly located in the GC at basal level and during infection, ST2+ cells were found in the red pulp and increased both in the red pulp and the marginal zone during infection, suggesting an influx or activation of both myeloid and lymphoid cells.

Data obtained by flow cytometry indicated a significant influx of B lymphocytes in the spleen of wt BALB/c mice until Day 60, which was correlated to PD-1 mRNA expression, whereas T cells remained at steady levels over time. Interestingly, PD-1 expression was less expressed in BALB/c ST2-deficient mice, who had lower parasite burdens. Interaction of the inhibitory receptor PD-1 with its ligand B7-H1 was shown to induce T cell anergy and apoptosis, thereby playing a key role in the induction of exhaustion, which can be targeted to restore CD8+ T cell functions towards pathogen elimination, including *Leishmania* [17–19]. We hypothesize that IL-33 could contribute to the induction of this pathway.

The recruitment of B lymphocytes during infection in wt BALB/c mice has been proposed to explain the inadequate immune response observed in this organ. Indeed, various models have yielded convicting data showing that (i) B cell depletion enhanced resistance of BALB/c mice to *L*. *mexicana* and *L. tropica* [20], and (ii) C57BL/6 B-cells deficient mice had a boosted capacity to resist to *L. donovani* infection [21]. B cells down-modulation of T cell response could occur either by IgM polyclonal activation together with activation of complement, or through IL-10 production by B cells in the spleen [22]. Although IL-10 induction paralleled the influx of B cells in our BALB/c mice (not shown), it was not correlated to that of IL-33 and was not reduced in ST2-deficient mice. Additionally, B cell recruitment did not increase in rIL-33-treated mice, thus it was not possible to reconcile IL-33 levels and IL-10 production by B cells in the spleen.

Repeated treatment of BALB/c mice with rIL-33 resulted in the long term in a complete exhaustion of the immune response, with a dramatic decrease of lymphoid cell recruitment, as well as of GC area and splenomegaly, thus worsening the natural outcome of infection in these mice. This reduced cell recruitment in rIL-33-treated mice was associated with a significantly lower expression of the B- and T cells-chemoattractant chemokines CXCL13 and CCL19, respectively (*P* < 0.001), as well as a dramatic decrease of Th1 cytokines (mainly IL-12, IFN-γ) which was nicely correlated to the decrease of CD4+ T cells. Such an abolition of responsiveness is also observed in heavily infected dogs [23] and in humans. Taken together, these findings suggest that IL-33 supplementation increased the splenic parasite burdens through impairment of Th1 effectors, thus aggravating the normal course of the disease in sensitive animals, leading to immune exhaustion in the spleen.

Parasite loads were better controlled in BALB/c ST2-deficient mice, suggesting a more efficient immune response. These results are in line with recent results obtained in a model of *L. infantum* visceral leishmaniasis [24], but also with other models of infection. In septic arthritis, Staurengo-Ferreari et al. [25] reported that ST2 deficiency shifted the immune balance toward a Th1 immune response contributing to eliminating the infection *via* NO enhanced production by neutrophils and macrophages. However, in our model, ST2-deficient mice had only a moderate induction of key effectors of the anti-*Leishmania* response, mainly IL-12; this is probably due to the intrinsic immunosuppressive effect of *L. donovani*. As already reported, the consequences of ST2 deficiency, and thus the effects of IL-33, may differ according to the pathogen and the micro-environment [6]. In a model of cryptococcosis, a reduction of IL-5 and IL-13 production by Th2 cells was observed in the absence of ST2 signaling and led to better control of the fungus *Cryptococcus neoformans*, despite no difference in the level of expression of IFN-γ [26]. In a murine model of experimental cerebral malaria induced by *Plasmodium berghei* ANKA, ST2 deficiency had a protective effect on the cognitive defects induced by excessive inflammation in the brain in relation with high levels of IL-1β induced by IL-33 [27]. Interestingly, we also observed a significant reduction of IL-1β induction in the spleen of ST2-KO mice, while this cytokine is described as an active pro-inflammatory effector that could contribute to inefficient inflammation in this organ.

Taken together, we observed some similarities in IL-33 effects in the spleen, as previously observed in the liver, i.e. (i) an absence of organomegaly in ST2-KO mice compared to wt, (ii) a dramatic decrease of cell attraction in rIL-33-treated BALB/c mice also associated with an absence of organomegaly and an abolition of Th1 cytokines (and others). However, the impact of ST2 deficiency had a markedly superior effect in boosting Th1 cytokine induction in the liver than in the spleen, probably in relation with the intrinsic mixed Th1/Th2 microenvironment in the liver and the high number of myeloid cell infiltrates in this organ.

With regard to B6 mice, an early induction of Th1 cytokines (IFN-γ, IL-12, TNF-α and IL-1β) was observed in IL-33-KO mice, ensuring early control of the splenic parasite burdens, which remained at low levels over the study period, and was associated with a significantly higher recruitment of NK cells, CD4+ and CD8+ T cells, but also B cells. Overall, B6 mice had only moderate splenomegaly and better controlled the parasite burdens than BALB/c mice. The supplementation of B6 mice with rIL-33 had little effect on cell recruitment and on cytokine expression at an early time point, while parasite loads appeared to be better controlled. However, parasitic loads increased over time, being maximal on Day 60, thus restoring a sensitive profile, whereas they significantly decreased in untreated mice. Unlike BALB/c mice, B6-treated mice kept an apparently efficient Th1 response (overall unmodified cytokines and iNOS, except a transient decrease if IFN-γ on Day 30), but underwent a strong induction of TGF-β and FoxP3, indicating a probable activation of Treg cells, as confirmed by immunohistological staining of tissue sections, which is in agreement with the known role of IL-33 on the activation of Treg cells [7], and would explain parasite persistence in the spleen. This increase of the Treg pathway was associated with a concomitant increase of Th2 cytokines (IL-5 and IL-13), as observed in several other models of infection and inflammatory diseases [6, 7, 28].

Overall, B6 and BALB/c mice showed a distinct splenic response to *L. donovani* infection, which was far better controlled by B6 mice, and associated with lower expression of IL-33 (at least double) and its receptor at basal level and during the course of disease. In both mouse strains, the lack of IL-33 or ST2 gene, respectively, promptly improved the parasite burden as well as in the long term. However, treatment with rIL-33 exerted divergent effects with early control and a burst of parasite growth, in B6 and BALB/c mice, respectively, and inverse effects in the long run. This suggests that IL-33 could contribute to improve the outcome in a Th1-biased genetic background, at an early stage of disease, while it could aggravate the disease in a Th2-biased background. Bourgeois et al. [29] reported that IFN-γ production by splenic iNKT cells from B6 mice could be induced by IL-33, irrespective of prior priming with α-galactoside ceramide (α-GC), while iNKT from BALB/c mice induced both IFN-γ and IL-4. This could explain the higher susceptibility of BALB/c mice driven by IL-33 during leishmaniasis.

Whether activation of Th1 pathways is beneficial or deleterious for the host is highly dependent on the model of infection and the target tissue. In brain infections, excessive inflammatory response leads to tissue damage and possible sequelae [30, 31]. During visceral leishmaniasis, splenic excessive inflammatory response can be associated to lack of control of parasite multiplication [32], but some studies in infected dogs showed a negative correlation between Th1 cytokines induction and parasite loads [23], highlighting the importance of Th1-driven effectors in the pathophysiological process. We hypothesize that IL-33 could contribute to splenic exhaustion and atrophy of germinal centers observed in infected humans, by decreasing Th1 effectors [32].

## Conclusions

Overall, this study provides a consistent body of data demonstrating that IL-33 plays a key role in the outcome of *Leishmania* infection in the spleen, though it had different effects in BALB/c and C57BL/6 mice. In susceptible BALB/c mice, IL-33 aggravates cell exhaustion possibly through PD-1 stimulation, while in C57BL/6 mice it has little effect in early stage of infection, but it is capable to elicit a Th2 response and reverse resistant phenotype towards susceptibility in the long term, possibly through Treg-induced pathways. In this context, strategies of blocking the action of IL-33 could be considered in therapeutics in order to boost the anti-parasite immune response in susceptible or immunocompromised patients.

## Supplementary information

**Additional file 1: Figure S1.** Quantification of cytokines levels in the serum of BALB/c mice infected with *L. donovani*. Quantification of IL-1 (**a**), IFN-β (**b**), CXCL-10 (**c**) and TNF-α (**d**) at day 15, 30 and 60 post-infection in BALB/c wt and ST2-KO mice by flow cytometry using a bead assay (FlowCytomix®), and quantification of IL-4 (**e**), IL-5 (**f**), and IL-13 (**g**) in BALB/c mice treated with IL-33 or untreated. Data show results from 4 to 7 mice per group (*P < 0.05; Mann-Whitney test).

**Additional file 2: Figure S2.** Quantification of cytokine levels in the serum of B6 mice infected with *L. donovani*. Quantification of TNF- (**a**) and IFN-α (**b**) in B6 wt and IL-33 KO mice at day 15 post-infection using an ELISA assay (R&D Systems). Quantification of IL-4 (**c**), IL-5 (**d**), IL-13 (**e**) and IL-1β at day 15, 30 and 60 post-infection in B6 mice treated with IL-33 or untreated (wt) mice by flow cytometry using a bead assay (FlowCytomix®). Data show results from 4 to 7 mice per group (*P < 0.05; Mann-Whitney test).

## Data Availability

Data supporting the conclusions are included within the article and its additional files. Detailed data are available upon request.

## Abbreviations

B6: C57BL/6
GC: germinal centers
HES: hematoxylin eosin saffran
IL-33: interleukin-33
IL-33-KO: IL-33 knock out
iNOS: inducible NO synthase
MFI: mean fluorescence intensity
rIL-33: recombinant IL-33
SEM: standard error of the mean
ST2-KO: ST2 knock out
VL: visceral leishmaniasis
wt: wild type
