# Supplementary figures and images for: IL-33/ST2 axis is involved in disease progression in the spleen during Leishmania donovani infection
Source: Parasit Vectors. 2020 Jun 22;13:320. doi: 10.1186/s13071-020-04190-3 (PMC7310124; doi:10.1186/s13071-020-04190-3)

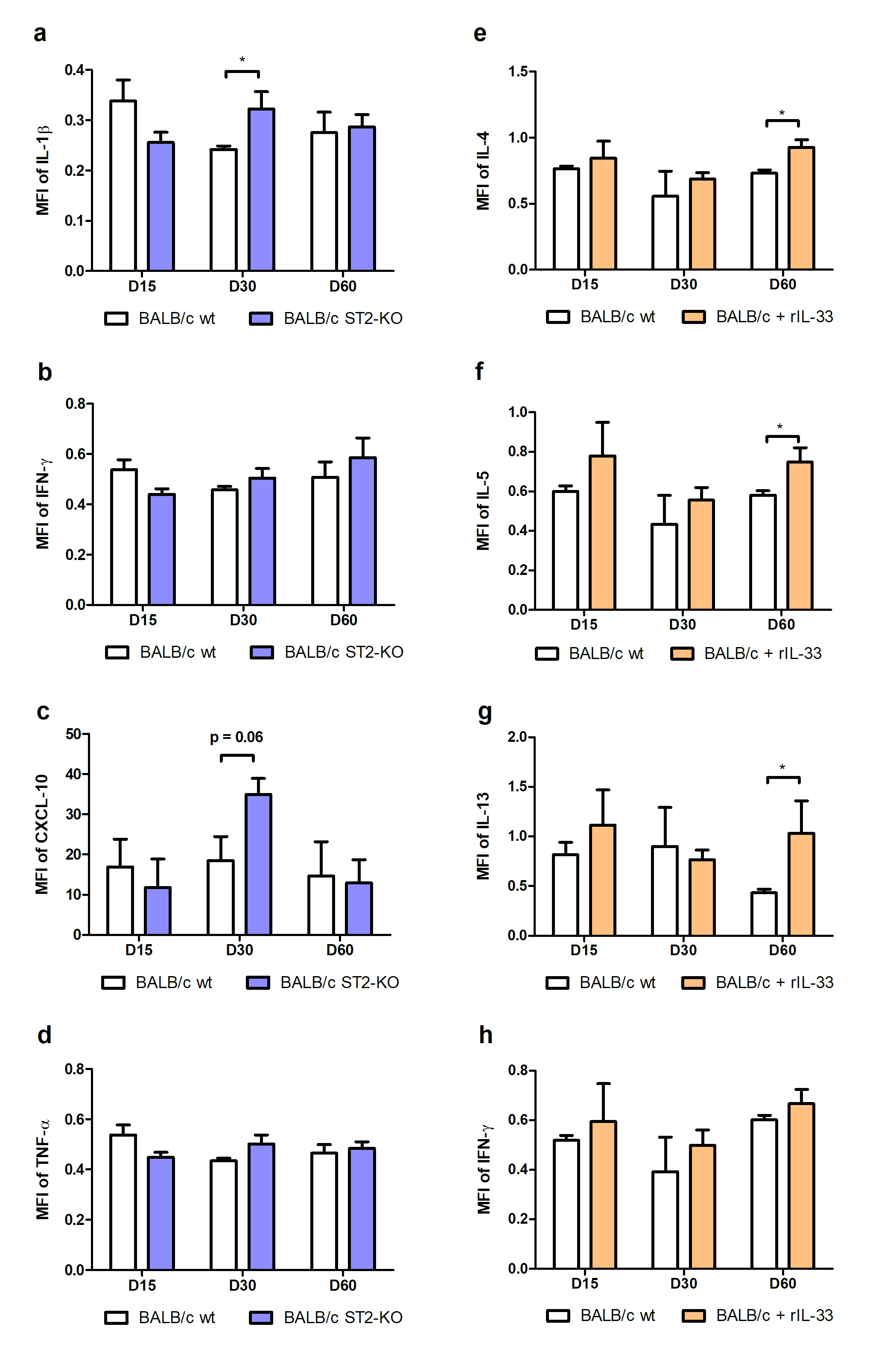

Supplement: Supplementary file 1 — Additional file 1: Figure S1. Quantification of cytokines levels in the serum of BALB/c mice infected with L. donovani. Quantification of IL-1 (a), IFN-β (b), CXCL-10 (c) and TNF-α (d) at day 15, 30 and 60 post-infection in BALB/c wt and ST2-KO mice by flow cytometry using a bead assay (FlowCytomix®), and quantification of IL-4 (e), IL-5 (f), and IL-13 (g) in BALB/c mice treated with IL-33 or untreated. Data show results from 4 to 7 mice per group (*P < 0.05; Mann-Whitney test). [file 13071_2020_4190_MOESM1_ESM.tif]

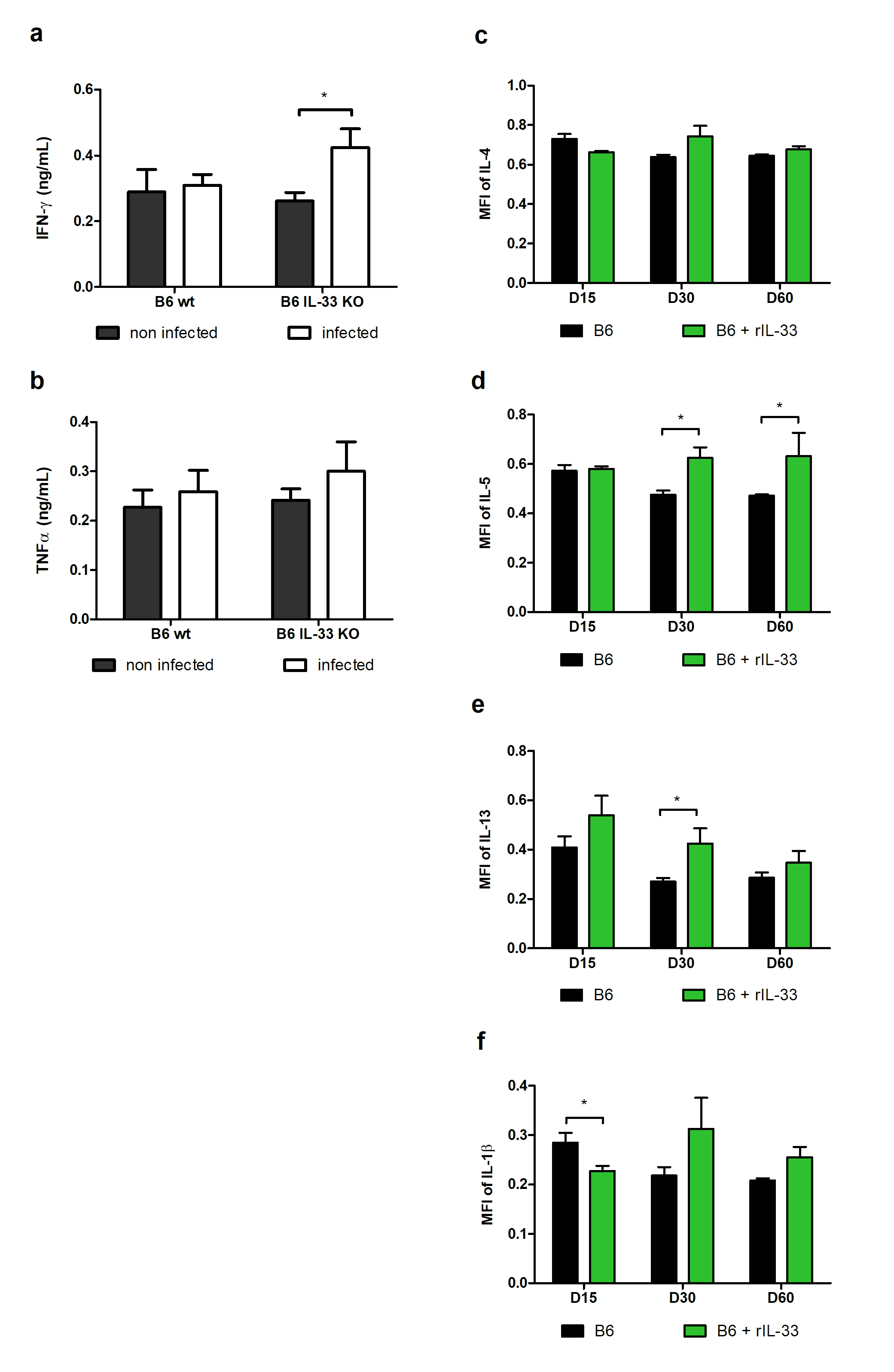

Supplement: Supplementary file 2 — Additional file 2: Figure S2. Quantification of cytokine levels in the serum of B6 mice infected with L. donovani. Quantification of TNF- (a) and IFN-α (b) in B6 wt and IL-33 KO mice at day 15 post-infection using an ELISA assay (R&D Systems). Quantification of IL-4 (c), IL-5 (d), IL-13 (e) and IL-1β at day 15, 30 and 60 post-infection in B6 mice treated with IL-33 or untreated (wt) mice by flow cytometry using a bead assay (FlowCytomix®). Data show results from 4 to 7 mice per group (*P < 0.05; Mann-Whitney test). [file 13071_2020_4190_MOESM2_ESM.tif]
